# Fibulin‐2: A potential regulator of immune dysfunction after bone trauma

**DOI:** 10.1002/iid3.846

**Published:** 2023-05-05

**Authors:** Shidan Li, Hao Jiang, Shaochuan Wang, Youbin Li, Debin Guo, Jijie Zhan, Qiaohui Li, Hao Meng, Ankang Chen, Limin Chen, Xiaoyan Dai, Xiaoming Li, Wei Xing, Lei Li, Jun Fei

**Affiliations:** ^1^ Department of Orthopaedics, State Key Laboratory of Trauma, Burn and Combined Injury, Daping Hospital Army Medical University Chongqing People's Republic of China; ^2^ Department of Orthopaedics Affiliated Hospital of Southwest Medical University Luzhou People's Republic of China; ^3^ Department of Emergency, Daping Hospital Army Medical University Chongqing People's Republic of China; ^4^ Department of Cancer Center, Daping Hospital Army Medical University Chongqing People's Republic of China; ^5^ Department of Military Traffic Injury Prevention, Daping Hospital Army Medical University Chongqing People's Republic of China; ^6^ Department of Stem Cell and Regenerative Medicine, State Key Laboratory of Trauma, Burn and Combined Injury, Daping Hospital Army Medical University Chongqing People's Republic of China

**Keywords:** bone trauma, fibulin‐2, immunity, transgenic mouse

## Abstract

**Objectives:**

To reveal the relationship between the fibulin‐2 protein and immune dysfunction after bone trauma.

**Methods:**

Individuals who were admitted to the study were divided into a bone trauma group, a recovered from bone trauma group and a volunteer without bone trauma group based on the reason for admission. Fibulin‐2 levels in the three groups were compared. *Fibulin‐2*‐knockout (*fibulin‐2* ^−/−^) mice and wild‐type (WT) mice were used to detect susceptibility to infection. Hematoxylin and eosin (HE) staining and immunohistochemical staining were employed to observe pathological changes in each organ from *fibulin‐2* ^−/−^ mice and WT mice.

**Results:**

In total, 132 patients were enrolled in this study. The fibulin‐2 level in the bone trauma group was lower than that in the recovered bone trauma group (3.39 ± 1.41 vs. 4.30 ± 1.38 ng/mL, *t* = 2.948, *p* < .05) and also lower than that in the volunteers without bone trauma group (3.39 ± 1.41 vs. 4.73 ± 1.67 ng/mL, *t* = 4.135, *p* < .05). *Fibulin‐2* ^−/−^ mice are more prone to infection. Compared with those in WT mice, spleen function and thymus function in *fibulin‐2* ^−/−^ mice were impaired. Immunohistochemical staining revealed that compared with those in WT mice, significantly fewer CD4+ T cells, CD8+ T cells, and CD19+ B cells were noted in the spleen and thymus of *fibulin‐2* ^−/−^ mice.

**Conclusions:**

The plasma fibulin‐2 level was lower in patients with bone trauma. Decreased fibulin‐2 is associated with immune dysfunction after bone trauma.

## Highlights

- Plasma fibulin‐2 is lower in patients with bone trauma.
- Fibulin‐2 deficiency is more prone to infection.
- The function of spleen and thymus in *fibulin‐2* ^−/−^ mice are impaired.
- The CD4+ T cells, CD8+ T cells, and CD19+ B cells are fewer in *fibulin‐2* ^−/−^ mice.

## Graphical abstract

This study may provide both a basis for in‐depth exploration of the mechanism by which the body is prone to secondary infection after bone trauma and novel ideas for further research and development of immunomodulatory drugs.

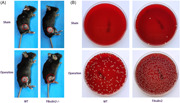

## 1 INTRODUCTION

Bone trauma refers to the destruction of the structural integrity of bone tissue or dysfunction of the skeleton caused by mechanical factors on the human body. With socioeconomic development, the overall incidence of bone trauma continues to increase.^1^, ^2^ According to the literature, the hospitalization rate for bone trauma patients increased from 0.31% to 0.57% between 2009 and 2016, with an annual growth rate of 9.1%^3^, ^4^ in China, resulting in a subsequent increase in economic and social burdens.^5^ Patients with bone trauma often experience hemorrhagic shock due to severe bleeding. As a result, some patients die in the prehospital emergency phase.^6^, ^7^ Even though some patients survive the emergency phase, they still need to overcome traumatic stress, including disorders of the internal environment and immune dysfunction. More seriously, some patients are prone to secondary infections due to immune dysfunction after bone trauma.^8^ Notably, 4%–64% of patients with open long bone trauma will develop an infection.^9^ As an increasing number of bacteria are resistant to antibiotics, the efficacy of antibacterial drugs in the treatment of bone infections is decreasing.^10^ If an early bone infection cannot be effectively controlled in a timely manner, it will progress to osteomyelitis, which is complicated by bone destruction.^11^, ^12^ Solving immune dysfunction after bone trauma may help decrease the incidence of infection.^13^

Studies have shown that damage to local tissues may lead to the release of inflammatory mediators to promote inflammatory responses after bone trauma. Simultaneously, inflammatory suppressive responses are also triggered in local damaged tissues. However, a dynamic balance between inflammation‐promoting and inflammation‐suppressing responses of the body is difficult to achieve, resulting in disordered immune homeostasis.^14^ Specific immunity is the third line of defence in human immunity, and the suppression of specific immunity is an important feature of posttraumatic immune dysfunction. Specific immunity is mainly composed of immune organs such as the thymus and spleen and immune cells such as lymphocytes. Immune organs are important sites for the production of immune cells, maturation of immune cells, and execution of immune functions. Among the immune cells, CD4+ T lymphocytes are important helper T lymphocytes that assist in humoural immunity and cellular immunity; CD8+ T cells are cytotoxic T lymphocytes with specific killing activity; and CD19+ B cells play an important role in the humoural immune response.^15^, ^16^ Previous studies have reported that the number of T cells and B cells in the body is reduced, and immune function is impaired. The depletion of immune cells and damage to immune organs will lead to persistent low immune function and increased susceptibility to secondary infection.^17^, ^18^ However, the mechanism is still unclear.

Extracellular matrix (ECM) proteins can regulate the release of inflammatory mediators and play important roles in the process of immune dysfunction after trauma.^19^, ^20^ As a secreted protein, fibulin‐2 is a constituent of the ECM.^21^ As reported in previous studies, fibulin‐2 is closely related to posttraumatic skin healing and is involved in the tumor immune process.^22^, ^23^ Additionally, in our previous study, we found that fibulin‐2 expression was increased in the plasma of infected patients, demonstrating that fibulin‐2 is associated with infection.^6^ Based on the above reasons, we speculate that fibulin‐2 may be associated with posttraumatic immune dysfunction. To explore the relationship between fibulin‐2 and bone trauma and posttraumatic immune dysfunction, we collected blood samples from patients with bone trauma (bone trauma group), patients who recovered from bone trauma during the same time period (recovered from bone trauma group) and volunteers without bone trauma. We found that the fibulin‐2 level in the plasma of the bone trauma group was lower than that in the plasma of the recovered bone trauma group and also lower than that in volunteers. To further explore the clinical significance of reduced fibulin‐2 levels, we constructed *fibulin‐2* gene‐knockout mice using CRISPR/Cas9 technology and evaluated the effect of *fibulin‐2* gene deletion. Fibulin‐2‐knockout mice were more susceptible to secondary infection, the spleen function and thymus function of knockout mice were impaired, and the B cells and T cells were correspondingly reduced, thus demonstrating that fibulin‐2 is associated with immune dysfunction after bone trauma. This study may provide a basis for the in‐depth exploration of the mechanism by which the body is prone to secondary infection after bone trauma and provides novel ideas for further research and development of immunomodulatory drugs.

## 2 MATERIALS AND METHODS

### 2.1 Main reagents

The following reagents were used: mouse tail genotype rapid identification kit and 2× Taq Master Mix (Beyotime Biotech); polymerase chain reaction (PCR)‐related reagents (TaKaRa); primers and DNA maker (Beijing Bioned Gene Technology Co., Ltd); agarose (Sigma); Human Fibulin‐2 enzyme‐linked immunosorbent assay (ELISA) Kit (CUSABIO) and hematoxylin staining solution, rabbit anti‐mouse primary antibodies for CD4, CD8, and CD19, HRP goat anti‐rabbit secondary antibody, and DAB chromogenic reagent (Wuhan Servicebio Technology Co., Ltd.).

### 2.2 Collection of clinical samples and clinical information

This study was approved by the Ethics Committee of the Daping Hospital (approval number: Medical Research Review (2021) NO 07). Patients who agreed to voluntarily participate between January 2021 and December 2021 were selected as the study subjects. The inclusion criteria were as follows: (1) patients with bone trauma and (2) patients who visited our hospital for re‐examination after recovering from bone trauma. The exclusion criteria were as follows: (1) patients with tumors and tuberculosis, (2) patients with incomplete medical records, (3) patients younger than 18 years old or older than 70 years old, (4) patients who refused to participate in this study, (5) patients with pathological fractures, (6) patients with immune diseases or who were using immunosuppressive drugs, and (7) patients with infection. The following information was collected: (1) sex, (2) age, (3) clinical diagnosis, (4) personal history of smoking and drinking, (5) comorbidities such as hypertension and diabetes, and (6) clinical test indicators: d‐dimer, hemoglobin, C‐reactive protein, alanine aminotransferase (ALT), aspartate aminotransferase (AST), urea nitrogen, creatinine concentrations, and neutrophil percentages.

### 2.3 Diagnostic criteria for bone trauma

In this study, bone trauma was comprehensively diagnosed by senior physicians based on each patient's medical history, symptoms, signs, and imaging evidence. Patients with a clear history of trauma, local manifestations of pain, swelling, or dysfunction after injury, and disruption of bone continuity detected by X‐ray, computed tomography (CT), or magnetic resonance imaging (MRI) were diagnosed with bone trauma.^24^, ^25^ All clinicians involved in the diagnosis of bone trauma did not know the fibulin‐2 levels of the patients.

### 2.4 Fibulin‐2 detection

Blood samples were collected from the included subjects within 12 h after admission. Blood samples (1 mL) were collected using heparin‐containing anticoagulated blood collection tubes BD Biosciences and stored in a refrigerator at 4°C. The blood samples were centrifuged at 4000*g* for 5 min within 6 h to obtain plasma. The plasma fibulin‐2 level was measured using an ELISA. Each sample was measured twice, and the average was taken as the final concentration. The individuals responsible for measuring fibulin‐2 did not know the grouping of the patients.

### 2.5 Experimental animals

*Fibulin‐2* ^−/−^ mice were prepared and provided by Cyagen Biosciences Co., Ltd. [SCXK (Guangdong) 2018‐0032]. The mice were housed at the Experimental Animal Centre of Army Medical University in accordance with the requirements for specific pathogen‐free‐grade animals [SYXK (Chongqing) 2017‐0005]. All experimental procedures were approved by the Animal Ethics Committee of Army Medical University (approval number: AMUWEC 20212163).

### 2.6 Obtaining and identifying positive mouse strains

The F0 generation of female mice was bred with C57BL/6 wild‐type (WT) male mice to obtain the first generation of mice, denoted as F1. Tails were used for genome extraction. The genotypes of F1 generation mice were identified by PCR (Primer‐F1 and Primer‐R1, see Table 1) and genome sequencing. The *fibulin‐2*‐knockout F1 generation of heterozygous male mice (*fibulin‐2* ^−/−^) was crossed with WT female mice to obtain the second generation of mice, denoted F2. The genotypes were identified (Primer‐F1 and Primer‐R1, see Table 1). Then, male and female F2 *fibulin‐2* ^−/−^ mice were crossed to obtain the third generation of mice, and genotype identification was performed (Primer‐F1, Primer‐F2, and Primer‐R1, see Table 1). The PCR conditions were as follows: initial denaturation at 95°C for 3 min; 35 cycles of 95°C for 15 s, 60°C for 15 s, and 72°C for 3 min; and extension at 72°C for 5 min.

**Table 1:** Sequences of primers used for mouse genotype identification.

Primer sequences	Sequence information	Product length (bp)
Primer‐F1	*ATAGGCCGTATGTATGGAAGT*	517
Primer‐F2	*CCACCGAACCTGGAAGGAATA*	785
Primer‐R1	*AATGTAAGCTGTGACTGAGAGAG*	

### 2.7 Establishment of a bone infection model

*Fibulin‐2* ^−/−^ mice and WT mice (6–8 weeks, 18–22 g) born in the same litter were randomly divided into a surgery group and a sham‐operation group. A 1‐cm incision was made on the lateral side of the right hind femur, and then, the subcutaneous fascia and muscle were separated layer by layer to expose the femur. An electric drill was used to create a 0.5‐mm hole in the unilateral cortical bone of the middle femur, which was then filled with gelatine sponge. Mice in the surgical group were injected with 20 μL of *Staphylococcus aureus* (5 × 10^5^ colony forming unit) in the gelatine sponge, and mice in the sham‐operation group were injected with 20 μL of normal saline in the gelatine sponge. The subcutaneous tissue and skin were then sutured layer by layer using 4‐0 absorbable sutures. After 21 days, the skin near the incision was observed for redness, swelling, and pus.

### 2.8 Bacterial culture

Twenty‐one days after the surgery, the femurs were harvested. The skin and muscle tissues were removed, and the femurs were placed in 10 mL of normal saline. Subsequently, a bacterial suspension was obtained by sonication in an ultrasonic oscillator for 20 min. A 100‐μL bacterial suspension was inoculated evenly into blood agar medium. After incubation in a 35°C bacterial incubator for 24 h, bacterial growth was assessed.

### 2.9 Tissue sectioning and staining

*Fibulin‐2* ^−/−^ mice and WT mice from the same litter (6–8 weeks old) were anaesthetized using 4% chloral hydrate (intraperitoneal injection). The mice were killed by cervical dislocation. The heart, liver, spleen, lung, kidney, and thymus of each mouse were collected and fixed in 4% paraformaldehyde, dehydrated using an ethanol gradient, embedded in paraffin, and prepared into 4‐μm‐thick sections. After staining with hematoxylin and eosin (HE), the morphological differences in the viscera of the two groups of mice were assessed under a microscope. The spleen and thymus of mice were subjected to immunohistochemical staining (the dilutions of CD4, CD8, and CD19 antibodies were 1:500, 1:200, and 1:500, respectively, and the dilution of the secondary antibody was 1:200). Finally, we collected images with a high‐resolution scanner. Densitometry was performed with ImageJ software.

### 2.10 Data analysis

Statistical analysis was performed using SPSS 25.0 statistical software (IBM). Measurement data conforming to a normal distribution are expressed as the mean ± standard deviation, and the independent sample *t* test was used for comparisons between the two groups. Count data are expressed as the number of cases (%), and comparisons of rates were performed using the *χ* ^2^ test and Fisher's exact test. Two‐sided *p* < .05 indicated statistical significance.

## 3 RESULTS

### 3.1 Clinical characteristics of the included patients

**Figure 1:**
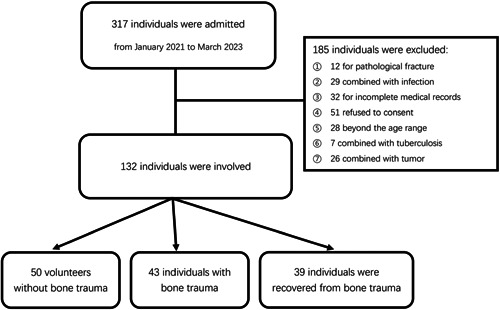
Flow chart of study population inclusion and exclusion.

On the basis of the inclusion and exclusion criteria, 317 patients were recruited. Thirty‐two patients had incomplete data, 12 patients had pathological fractures, 28 patients did not meet the age requirements, 51 patients refused to participate in this study, seven patients had combined tuberculosis, and 26 patients had tuberculosis. Twenty‐nine patients had combined tumors, and 29 patients had infections; ultimately, 132 patients were enrolled in this study. The patients were divided into a bone trauma group, a recovered from bone trauma group, and volunteers without bone trauma group based on their clinical diagnosis. The volunteers without bone trauma group included 50 individuals and bone trauma group included 43 patients, while 39 patients were included in the recovered from bone trauma group (Figure 1). Bone trauma was attributed to the following causes: traffic injury (*n* = 24), fall from height (*n* = 9), crush injury (*n* = 4), impact injury (*n* = 3), and other injury (*n* = 3). Bone trauma occurred at the following sites: limb fracture (*n* = 20), spine fracture (*n* = 7), rib fracture (*n* = 2), head fracture (*n* = 3), clavicle fracture (*n* = 2), pelvic fracture (*n* = 2), scapula fracture (*n* = 1), sternal fracture (*n* = 1), and multiple fracture sites (*n* = 5). The average age of patients with bone trauma was 52.56 ± 17.37 years, and the average age of patients who recovered from bone trauma was 52.77 ± 17.04 years. No significant difference in the age was found between the two groups. No significant difference in the number of patients with a history of smoking, drinking, diabetes, hypertension, or coronary heart disease was identified between the two groups (Figure 1 and Table 2).

**Table 2:** Demographic data of the enrolled patients.

	Volunteer	Bone trauma	Recovered	Statistical value 1	Statistical value 2
Number of patients	50	43	39		
Age (years)	32.13 ± 11.4	52.56 ± 17.37	52.77 ± 17.04	*t* = 6.791, *p* = .000	*t* = −0.053, *p* = .958
Sex, male (female)	26 (24)	28 (15)	22 (17)	*χ* ^*2*^ = 1.633, *p* = .201	*χ* ^*2*^ = .651, *p* = .420
Personal history					
Smoking history, *n* (%)	14 (28.0%)	15 (34.9%)	9 (23.1%)	*χ* ^*2*^ = .510, *p* = .475	*χ* ^*2*^ = 1.377, *p* = .241
Drinking history, *n* (%)	9 (18.0%)	8 (18.5%)	6 (15.4%)	*χ* ^*2*^ = .006, *p* = .940	*χ* ^*2*^ = .150, *p* = .669
Comorbidity					
Diabetes, *n* (%)	0	4 (9.3%)	5 (12.8%)	*p* = .042	*χ* ^*2*^ = .259, *p* = .611
Hypertension, *n* (%)	1 (2.0%)	7 (16.3%)	10 (25.6%)	*p* = .023	*χ* ^*2*^ = 1.091, *p* = .296
Coronary heart disease, *n* (%)	0	2 (4.7%)	1 (2.7%)	*p* = .211	*P* = 1.000
Systolic BP (mmHg)	125.5 ± 20.3	128.9 ± 22.9	131.6 ± 23.0	*t* = −0.759, *p* = .450	*t* = −0.825, *p* = .412
Diastolic BP (mmHg)	72.9 ± 11.2	76.7 ± 13.6	76.9 ± 13.5	*t* = −0.156, *p* = .123	*t* = −0.117, *p* = .907
d‐dimer (mg/L)	/	1149.3 ± 2234.1	745.3 ± 2089.9	/	*t* = 1.429, *p* = .154
Hemoglobin (g/L)	/	127.9 ± 21.5	129.9 ± 25.3	/	*t* = −0.683, *p* = .495
Percentage of neutrophils (%)	/	75.7 ± 11.8	73.1 ± 12.6	/	*t* = 1.743, *p* = .082
C‐reactive protein (mg/L)	/	40.5 ± 51.3	16.8 ± 35.5	/	*t* = 3.873, *p* = .000
ALT (U/L)	/	48.5 ± 91.8	46.5 ± 74.4	/	*t* = 0.190, *p* = .850
AST (U/L)	/	50.8 ± 113.3	48.2 ± 223.6	/	*t* = 0.146, *p* = .884
Urea nitrogen (mmol/L)	/	6.7 ± 4.3	14.8 ± 83.1	/	*t* = −0.781, *p* = .435
Creatinine (μmol/L)	/	92.3 ± 127.8	100.3 ± 123.1	/	*t* = −0.488, *p* = .626
Fibulin‐2 (ng/mL)	4.73 ± 1.67	3.39 ± 1.41	4.30 ± 1.38	*t* = 4.135, *p* = .000	*t* = 2.948, *p* = .004

Abbreviations: ALT, alanine aminotransferase; AST, aspartate aminotransferase; Bone trauma, patients with bone trauma group; BP, blood pressure; Recovered, patients recovered from bone trauma group; Statistical value 1, volunteer versus bone trauma; Statistical value 2, bone trauma versus recovered; Volunteer, volunteers without bone trauma group.

### 3.2 Plasma fibulin‐2 is lower in patients with bone trauma

**Figure 2:**
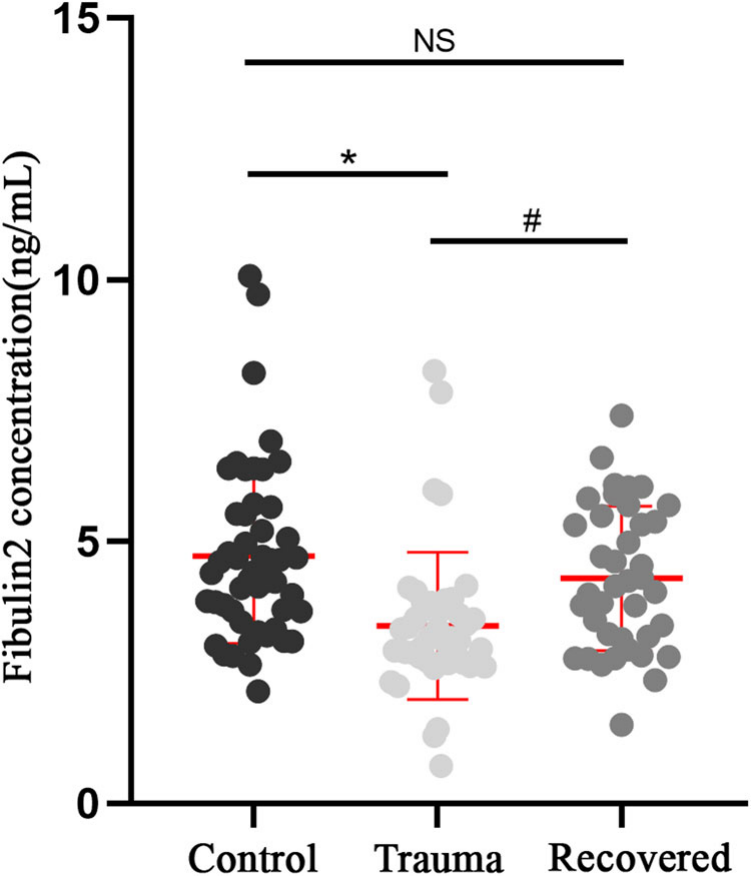
Plasma fibulin‐2 levels in different individuals. (Control group: volunteers without trauma; Trauma group: patients with bone trauma; Recovered group: patients recovered from bone trauma. Red bars represent the mean ± standard deviation; * and ^#^ *p* < .05, NS, no significance.

The fibulin‐2 level in patients with bone trauma was 3.39 ± 1.41 ng/mL, and the fibulin‐2 level in nonbone trauma patients was 4.30 ± 1.38 ng/mL. Plasma fibulin‐2 levels were lower in patients with bone trauma than in those without bone trauma; the difference was statistically significant. In addition, the fibulin‐2 level in patients with bone trauma was also lower than that in the volunteers without bone trauma group (3.39 ± 1.41 vs. 4.73 ± 1.67 ng/mL, *t* = 4.135, *p* < .05) (Figure 2).

### 3.3 *Fibulin‐2* ^−/−^ mice were constructed successfully

**Figure 3:**
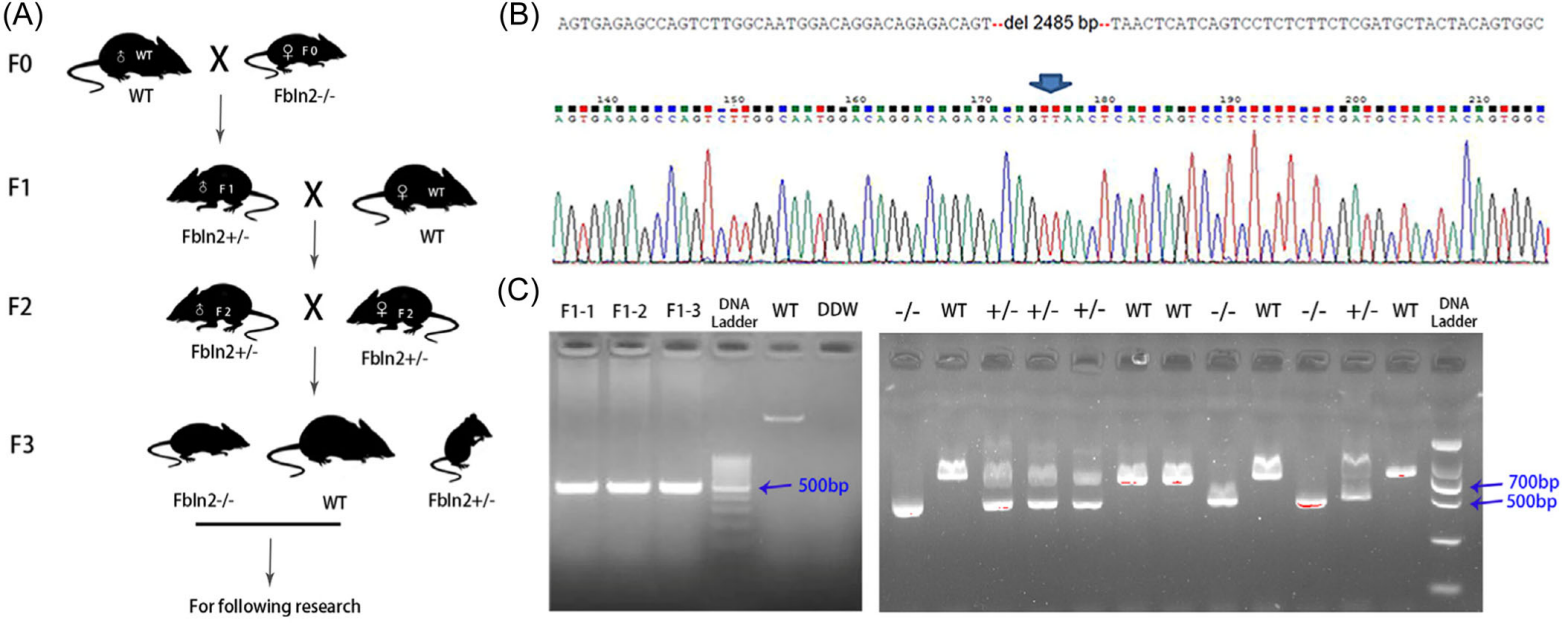
Breeding and identification of *fibulin‐2* ^−/−^ mice. (A) *Fibulin‐2* ^−/−^ mouse breeding strategy; F0, F1, F2, and F3 represent the primary, first‐, second‐, and third‐generation mice, respectively. (B) Genome sequencing results. The region indicated by the blue arrow in the figure is the gene deletion fragment. (C) PCR results for the offspring mice. PCR, polymerase chain reaction.

The mice were bred according to the method shown in Figure 3A. The genotypes of three F1 male mice were identified. The genome sequencing results (Figure 3B) showed a 2485‐bp gene deletion in the *fibulin‐2* gene in F1 male mice. The PCR results (Figure 3C) indicated that the length of the PCR product of three F1 male mice was shorter than that of the PCR product of the WT mice, confirming that the *fibulin‐2* gene was successfully knocked out. Then, F1 male mice were crossed with WT female mice. After obtaining the offspring, heterozygous mice were selected for female–male crossing, resulting in homozygous *fibulin‐2*‐knockout mice. As shown in Figure 3C, the PCR product for *fibulin‐2* ^−/−^ mice was 517 bp, that for WT mice was 785 bp, and those for *fibulin‐2* ^+/−^ mice were 517 and 785 bp, indicating that *fibulin‐2* gene‐knockout mice were successfully constructed.

### 3.4 *Fibulin‐2* ^−/−^ mice are more prone to secondary infection after bone trauma

**Figure 4:**
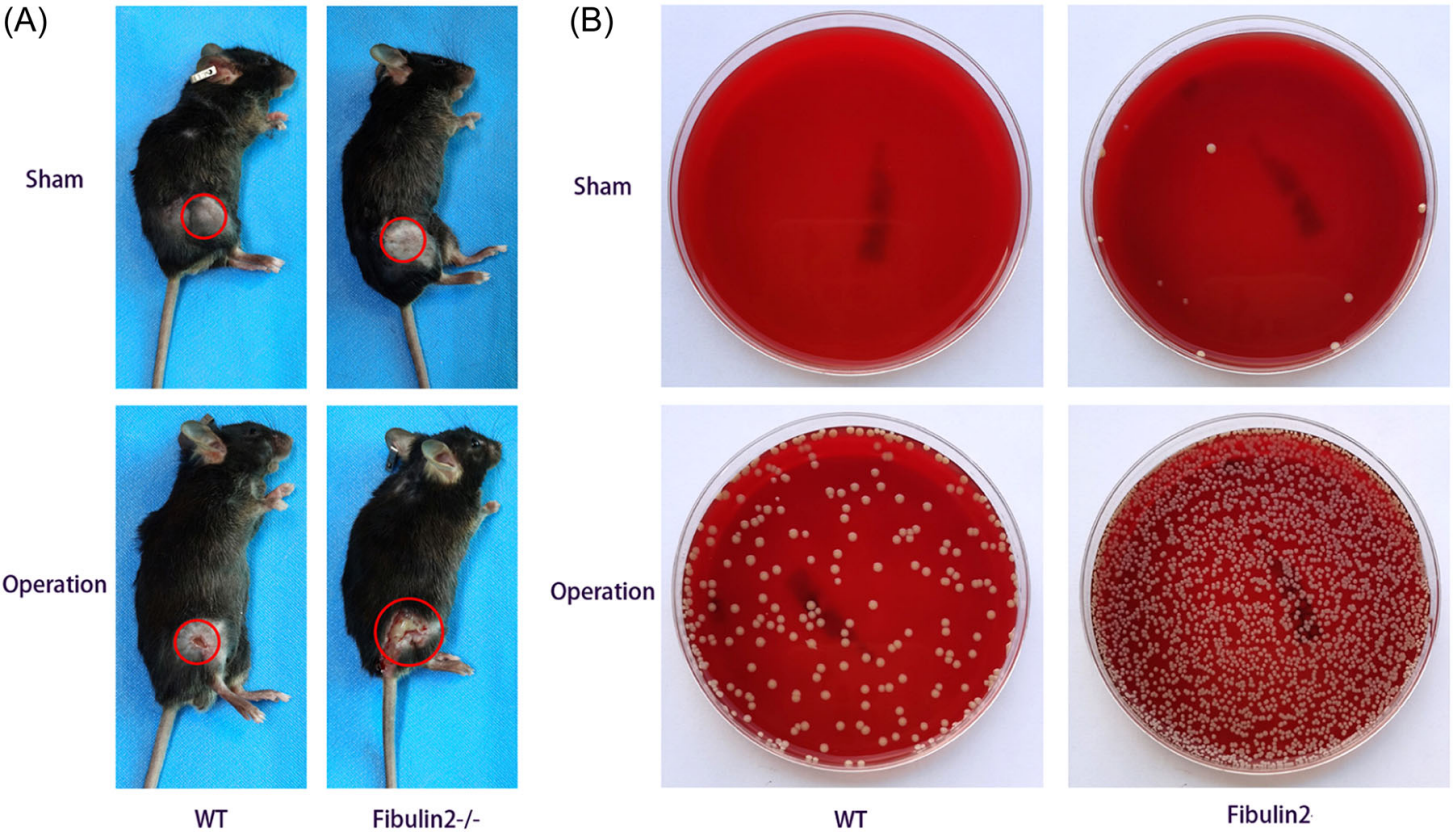
*Fibulin‐2* ^−/−^ mice are more prone to infection. (A) Postoperative skin healing; red circle indicates the postoperative wound; (B) bacterial cultures from mouse femurs.

To compare local skin healing among the mice after the operation, we found that the skin of the mice in the sham‐operation group healed well, with no local redness, swelling, or pus. For mice in the surgery group, the skin of WT mice did not completely heal, and local ulceration, redness, and swelling were observed, but no pus was noted. *Fibulin‐2* ^−/−^ mice had more extensive skin ulceration and more localized purulent exudate than WT mice (Figure 4A).

Then, the right femurs of the mice were collected for bacterial culture after surgery. The results showed no bacterial growth in the femurs of WT mice in the sham operation group, and a very small amount of bacterial growth was identified in the *fibulin‐2* ^−/−^ mice. In the surgery group, *S. aureus* was present in WT mice, but the colony‐forming units were fewer than those in *fibulin‐2* ^−/−^ mice (Figure 4A). Therefore, compared with WT mice, *fibulin‐2* ^−/−^ mice are more prone to secondary infection after bone trauma.

### 3.5 The function of spleen and thymus in *fibulin‐2* ^−/−^ mice are impaired

**Figure 5:**
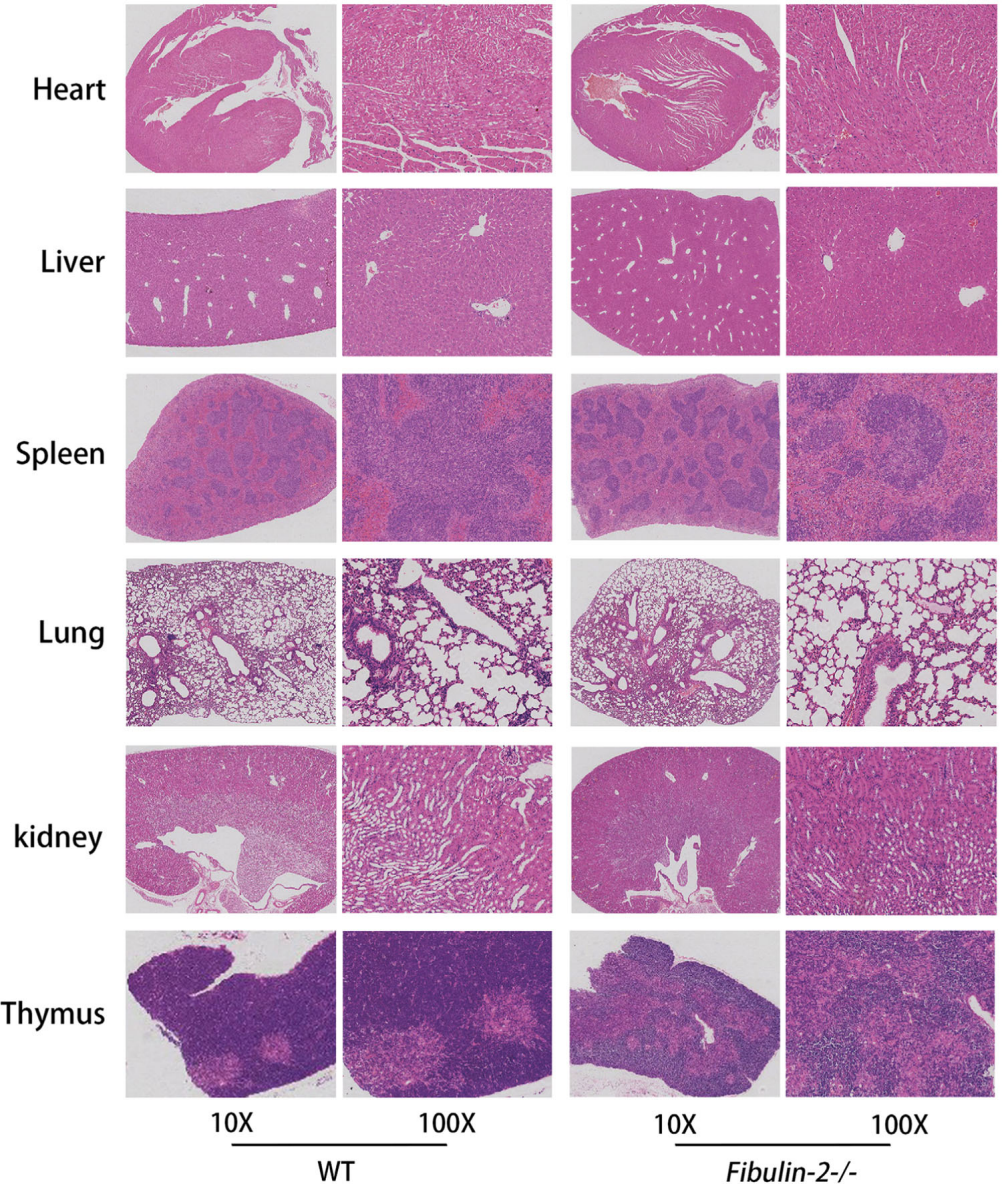
HE staining of various organs in mice. HE, hematoxylin and eosin.

To further explore why *fibulin‐2* ^−/−^ mice are more prone to secondary infection, we performed morphological observations of various organs from *fibulin‐2* ^−/−^ mice and WT mice born in the same litter. The results are shown in Figure 5.

#### 3.5.1 Heart

*Fibulin‐2* ^−/−^ mice and WT mice did not have significant myocardial thickening or cardiac hypertrophy. The myocardial fibers were long, with few branches, and were arranged regularly and tightly, and the gaps were small. No significant difference was observed between the two groups.

#### 3.5.2 Liver

HE staining revealed clear liver lobule structures, hepatic cords were arranged regularly with a radial shape, the morphology and structure of hepatocytes were normal, the nuclei were stained lightly, and the nucleoplasm was evenly distributed in a spherical shape. The central vein, venules, arterioles, and hepatic sinusoids were intact, with no inflammatory cell infiltration, and no hepatocyte necrosis was observed.

#### 3.5.3 Spleen

The spleens of WT mice had clear white and red pulp structures with obvious boundaries. The red pulp was distributed outside the white pulp and marginal zone. The marginal zone had a clear structure and separated the white pulp and red pulp; the white pulp was developed and had abundant splenic corpuscles and a distinct germinal center, and the splenic sinuses were not congested. The spleens of *fibulin‐2* ^−/−^ mice showed varying degrees of injury, with reduced white pulp and a slightly disordered structure. The boundary between white pulp and red pulp was relatively blurred, mostly in small patches scattered in the red pulp, the splenic corpuscles were reduced, and the germinal center was reduced.

#### 3.5.4 Lung

Overall, the alveolar structure of the two groups of mice was intact, the alveolar septum was normal, the alveolar cavity was clear, no exudate was noted, and the alveolar wall thickness was uniform. The bronchiolar epithelium was intact, and no obvious exudation was observed in the lumen. No significant neutrophil and monocyte infiltration and no congestion were observed in the alveolar septum.

#### 3.5.5 Kidney

The structure of kidney tissue was clearly visible in the two groups of mice. The glomerulus, capsule, tubules, proximal tubules, and distal tubules were structurally intact, with no edema and no necrosis. No inflammatory cell infiltration was found in the renal interstitium.

#### 3.5.6 Thymus

Clear boundaries between the cortex and medulla of the thymus in WT mice were observed. The boundary between the thymic cortex and the medulla of *fibulin‐2* ^−/−^ mice was blurred, and the cortical area was atrophied relative to the medullary area.

The results showed that compared with those of WT mice, the morphologies of the spleen and thymus of *fibulin‐2* ^−/−^ mice exhibited greater changes.

### 3.6 The CD4+ T cells, CD8+ T cells, and CD19+ cells are fewer in *fibulin‐2* ^−/−^mice

**Figure 6:**
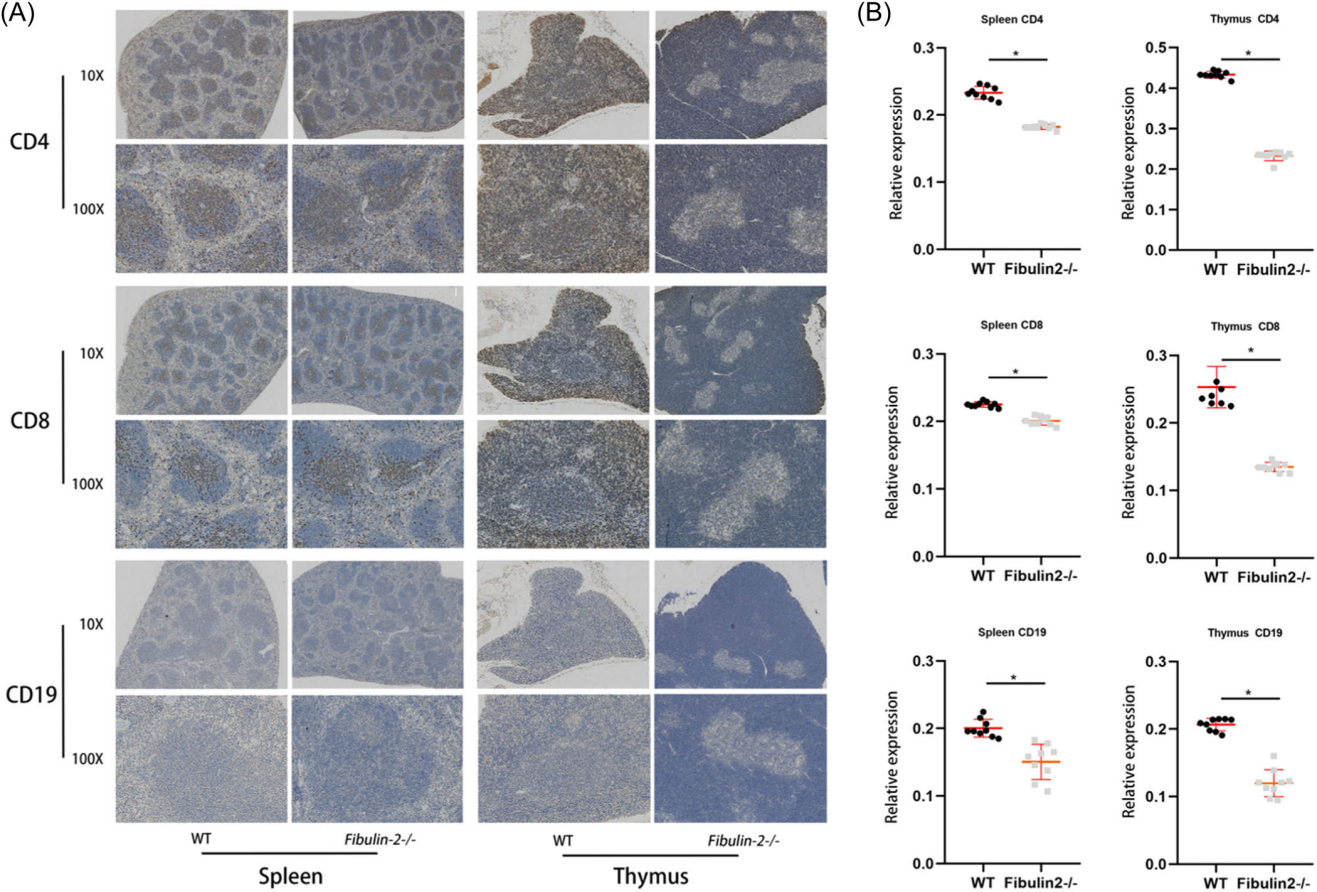
Detection of the CD4+ T cells, CD8+ T cells, and CD19+ cells in spleen and thymus. (A) Immunohistochemical staining of the spleen and thymus. (hematoxylin‐stained nuclei are shown in blue, and the corresponding positive antigen expression area is shown in brown). (B) Quantification of the immunohistochemical staining red bars represent the mean ± standard deviation; * *p* < .05). Fibulin‐2^−/−^ group: *Fibulin‐2*‐knockout mice; WT group, wild type mice.

To further assess spleen and thymus damage in *fibulin‐2* ^−/−^ mice, we performed an immunohistochemical analysis of immune cells in the spleen and thymus. We used CD4, CD8 and CD19 antibodies to label CD4+ T cells, CD8+ T cells, and CD19+ B cells, respectively. The results are shown in Figure 6. Compared with those in WT mice, the numbers of CD4+ T cells, CD8+ T cells, and CD19+ B cells in the spleen of *fibulin‐2* ^−/−^ mice were reduced, the difference was statistically significant (*p* < .05). In the thymus, WT mice had a large number of CD4+ T cells, CD8+ T cells, and CD19+ B cells; in comparison, in the *fibulin‐2* ^−/−^ mouse thymus, the numbers of CD4+ T cells, CD8+ T cells, and CD19+ B cells were significantly reduced and significantly lower than that in the WT mice (*p* < .05).

## 4 DISCUSSION

Fibulin‐2 belongs to the fibulin family and is a secreted protein mainly present in the ECM. Recent studies on fibulin‐2 mainly focus on tumor metastasis and cardiac remodeling; studies on fibulin‐2 and bone trauma are notably lacking.^22^, ^23^ Previous studies have reported that fibulin‐2 is closely associated with skin healing after bone trauma^22^, ^23^ We speculated that fibulin‐2 and bone trauma may be associated. To identify this possibility, we collected blood specimens from patients with bone trauma and patients who had recovered from bone trauma and measured fibulin‐2 levels in plasma. The results showed that compared with that in the plasma of patients in the non‐bone trauma group, the concentration of fibulin‐2 in the plasma of patients in the bone trauma group was lower. Because bone trauma usually has a clear cause of injury and the occurrence of bone trauma is accidental, fibulin‐2 cannot be used as a marker for predicting or diagnosing bone trauma, but fibulin‐2 may serve as a prognostic indicator of bone trauma. To further explore the role of fibulin‐2 in bone trauma, we successfully constructed *fibulin‐2* ^−/−^ mice and compared the pathological changes in various organs between *fibulin‐2* ^−/−^ mice and WT mice. The spleen and thymus in *fibulin‐2* ^−/−^ mice were damaged. Because the spleen and thymus are the main immune organs in the body, to further confirm whether immune function was also disrupted after tissue damage, we used immunohistochemical staining to label B cells and T cells in the spleen and thymus. The results showed fewer CD4+ and CD8+ T cells and CD19+ B cells in the spleen and thymus of *fibulin‐2* gene‐deficient mice than in the spleen and thymus of WT mice. Therefore, fibulin‐2 may be associated with impaired immune organ function after trauma.

In this study, when breeding mice, in the F1 generation mice obtained by crossing F0 generation *fibulin‐2* ^−/−^mice with WT mice, no male–female mice were obtained because all three F1 mice born in the first litter were males, and one F1 mouse born in the second litter was also male. Therefore, in this study, the F1 generation of mice could not be crossed to obtain offspring; therefore, the F2 generation of mice was obtained by crossing the F1 generation of mice with the WT mice. Male *fibulin‐2* ^+/−^ and female *fibulin‐2* ^+/−^ mice were selected and crossed to obtain *fibulin‐2* ^−/−^ mice and WT mice for subsequent experiments. Additionally, after obtaining *fibulin‐2* ^−/−^ mice, we mated male and female *fibulin‐2* ^−/−^mice and found that the female mice were infertile; as such, offspring *fibulin‐2* ^−/−^ mice could not be obtained through this method. Therefore, to obtain *fibulin‐2* ^−/−^ mice, *fibulin‐2* ^+/−^ mice were used for male–female crossing. *Fibulin‐2* ^−/−^ mice were confirmed through genetic identification and then used in subsequent experiments.

Because pathological sections can be used to observe damage to organs and tissues caused by diseases at the cellular level,^26^ in this study, pathological sections, HE staining, and immunohistochemical staining were used to analyze the effects of fibulin‐2 deficiency on various organs. The spleen and thymus are important immune organs in the body. The splenic parenchyma is composed of white pulp, red pulp, and a marginal zone. The white pulp is mainly composed of lymphocytes and is the main site for specific immunity. The marginal zone is dominated by B cells and is an important site for recognizing antigens and inducing immune responses. Immune cells are the basis of the immune functions of the spleen, which can exert specific immune functions in the body through T cell‐mediated cellular immunity and B‐cell‐mediated humoural immunity.^27^ The main function of the thymus is to produce T lymphocytes and participate in cellular immunity.^28^ The pathological results showed that in *fibulin‐2*‐knockout mice, both spleen function and thymus function were impaired, and the corresponding CD4+ and CD8+ T cells and CD19+ B cells, which perform immune functions, were reduced. Thus, fibulin‐2 may be a potential molecule associated with impaired immune organ function.

According to the literature, bone trauma leads to local tissue damage. Injured or dead cells release various injury‐related molecular patterns into the extracellular environment that act on pattern recognition receptors (e.g., Toll‐like receptors, purine receptors, protease‐activated receptors, and complement receptors) and activate these receptors to induce complex immune responses, resulting in immune dysregulation.^29^ The susceptibility of patients to infection increases after bone trauma, and some patients develop secondary infections, thereby prolonging their hospital stay and, in some cases, becoming life‐threatening. Previous studies have shown that within a few days after bone trauma, a considerable number of bone trauma patients will develop multiple organ dysfunction syndrome or even sepsis due to immune dysfunction, significantly increasing the mortality rate for bone trauma patients.^30^ However, the specific mechanism of immune disorders in patients after bone trauma remains unclear. Based on clinical cases, this study found that plasma fibulin‐2 levels in bone trauma patients were lower than those in non‐bone trauma patients. The knockout mouse model also revealed that *fibulin‐2* ^−/−^ mice had impaired spleen and thymus function and a corresponding decrease in the number of T cells and B cells. Therefore, fibulin‐2 is inferred to be associated with immune function after bone trauma. This study provides a theoretical basis for the study of immune dysfunction in patients with bone trauma and provides novel ideas for further improving the immune function of patients with bone trauma.

### 4.1 Limitations

This study used clinical data to determine whether the fibulin‐2 level in patients with bone trauma was reduced. Due to the small sample size, the effect of fibulin‐2 on the prognosis of patients with bone trauma was not further explored in clinical samples. Follow‐up studies could further explore secondary infections in patients with low or normal fibulin‐2 levels after bone trauma, and more clinical studies with larger sample sizes can be conducted to clarify the association of fibulin‐2 with concomitant infections in immune dysfunction after bone trauma. In addition, animal experiments were limited to pathological sections and immunohistochemical studies. The spleen function and thymus function of fibulin‐2‐deficient mice were found to be impaired, and the number of B cells and T cells was reduced. However, no further in vitro cell experiments or molecular mechanism experiments were conducted. More experiments are needed to demonstrate that reduced fibulin‐2 is a key factor in immune organ impairment after bone trauma. Additionally, this study revealed that the numbers of B cells and T cells in the spleen and thymus of *fibulin2* ^−/−^ mice were reduced; however, the relationship between fibulin‐2 and B cells and T cells was not further explored. Follow‐up experiments can use fibulin‐2 protein to stimulate B cells or T cells to observe the role of fibulin‐2 in the proliferation, differentiation, and maturation of immune cells.

## AUTHOR CONTRIBUTIONS

Shidan Li designed and performed most of the experiments, analyzed the data, and contributed to manuscript preparation. Hao Jiang, Shaochuan Wang, Youbin Li, Debin Guo, Jijie Zhan, Qiaohui Li, Hao Meng, Ankang Chen, Limin Chen, Xiaoyan Dai, and Xiaoming Li assisted with the experiments. Wei Xing revised the manuscript. Lei Li and Jun Fei conceived, designed, and supervised the project and revised the manuscript.

## CONFLICT OF INTEREST STATEMENT

The authors declare no conflict of interest.

## ETHICS STATEMENT

We have complied with all ethical regulations relevant to this research. Clinical specimens were collected from patients at Daping Hospital (Chongqing, China). The study followed the law and was approved by the Daping Hospital Clinical Ethics Committee (approval number: Medical research review (2021) No. 07). All specimens were collected with the informed consent of the patients. All animal experiments were performed in the Animal Laboratory Center of Third Military Medical University according to a protocol authorized by the Laboratory Animal Welfare and Ethics Committee of Third Military Medical University (approval number: AMUWEC 20212163).

## Data Availability

Data supporting the findings reported in this manuscript are available from the corresponding author upon reasonable request.
